# Papillary Thyroid Carcinoma with Fibromatosis/Fasciitis-Like/Desmoid-Type Stroma: Case Report of a Rare Subtype with Cytological and Molecular Study

**DOI:** 10.1007/s12105-024-01720-8

**Published:** 2024-10-22

**Authors:** Hosamadean Benghashir, Mahir Petkar, Rajen Goyal

**Affiliations:** Department of Laboratory Medicine and Pathology, Hamad Medical Corporation, Doha, Qatar

**Keywords:** Thyroid, Papillary thyroid carcinoma, Fibromatosis/fasciitis-like/desmoid-type stroma, Cytology, Molecular

## Abstract

**Background:**

Papillary thyroid carcinoma (PTC) with fibromatosis/fasciitis-like/desmoid-type stroma is a rare subtype of PTC,characterized by two distinct components: a classic papillary carcinoma component and a spindle cell proliferationresembling fibromatosis or nodular fasciitis. This stromal component adds a unique dimension to the tumor'spathology, making diagnosis more challenging and potentially leading to misclassification.

**Case presentation:**

We present a case of this rare entity which contributes to the growing body of literature by providing additionalmolecular data, which may shed light on the biological behaviour of the fibromatosis-like stroma and its relationshipwith the papillary carcinoma component. This case underscores the importance of recognizing this subtype, as itsspindle cell proliferation could be mistaken for a separate neoplasm or reactive process, resulting in inappropriatemanagement.

**Conclusions:**

Increased awareness of this entity will help pathologists avoid diagnostic pitfalls and guide clinicians in developingmore precise treatment plans, addressing both the malignant papillary component and the unique stromal features.This case further enriches the current understanding of the heterogeneity of PTC and highlights the need fortailored management strategies in rare subtypes.

## Case Presentation

**Fig. 1:**
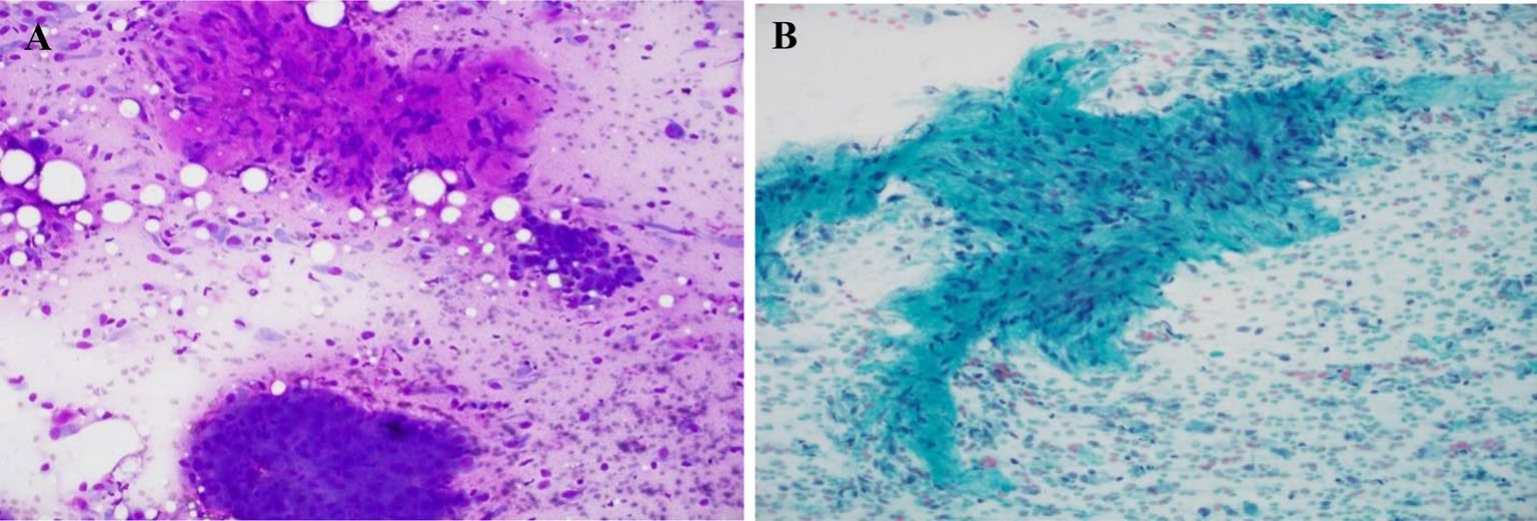
**A**&**B**. Fine-needle aspiration smears. Groups of epithelial cells showing highly atypical nuclear features with numerous elongated, single cells showing irregular nuclei and abundant cytoplasm (Romanowsky and Papanicolaou stains respectively)

A 41-year-old Nepalese female presented to Hamad General Hospital complaining of a thyroid lump for one week duration. Physical examination revealed a painful firm lump with no compression symptoms. Results of routine laboratory investigations, including thyroid function tests, were within normal ranges except for mild anemia. The patient had a smoking history but no history or family history of thyroid disease. Ultrasound imaging of the neck showed an enlarged thyroid gland with a complex right lobe nodule (designated TIRADS 4) and slightly enlarged cervical neck lymph nodes. Ultrasound guided fine-needle aspiration cytology (FNAC) of the thyroid nodule was performed. Cytology smears of the sample showed a lesion comprised of groups of epithelial cells showing highly atypical nuclear features with numerous elongated, single cells showing irregular nuclei but abundant cytoplasm. Epithelial and spindle cell features were both present. No necrosis was seen. The appearances were those of a malignant neoplasm that was difficult to categorize and that medullary carcinoma should be considered (Fig. 1).

The patient underwent a total thyroidectomy with central lymph node dissection. Intraoperatively, the right thyroid lobe was large and firm in consistency and was attached and tethered to trachea.

**Fig. 2:**
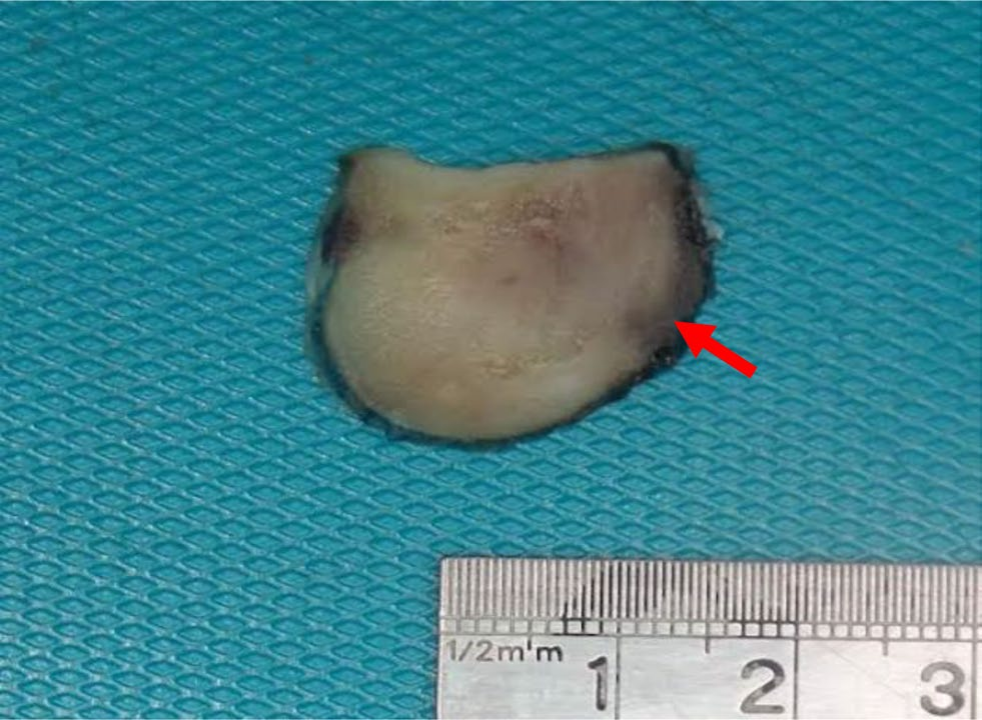
Gross picture of cross section of mass. The mass had an overall homogenous tan-grey firm solid appearance. Skeletal muscle is seen at the right periphery which shows gross/macroscopic erosion and infiltration by the mass (red arrow)

The total thyroidectomy and central node dissection specimens were sent to our pathology laboratory for processing. The thyroid weighed 53 g and the right lobe measured 5.6 × 3 × 2 cm. On gross examination, the outer surface of the lobe was nodular. The cut section of the right lobe revealed a lesion showing a diffuse grayish, white, fasciculated and trabeculated appearance occupying almost the entirety of the right lobe. Skeletal muscle was seen at the periphery and was eroded by the mass (Fig. 2). The remainder of the thyroid was grossly unremarkable.

**Fig. 3:**
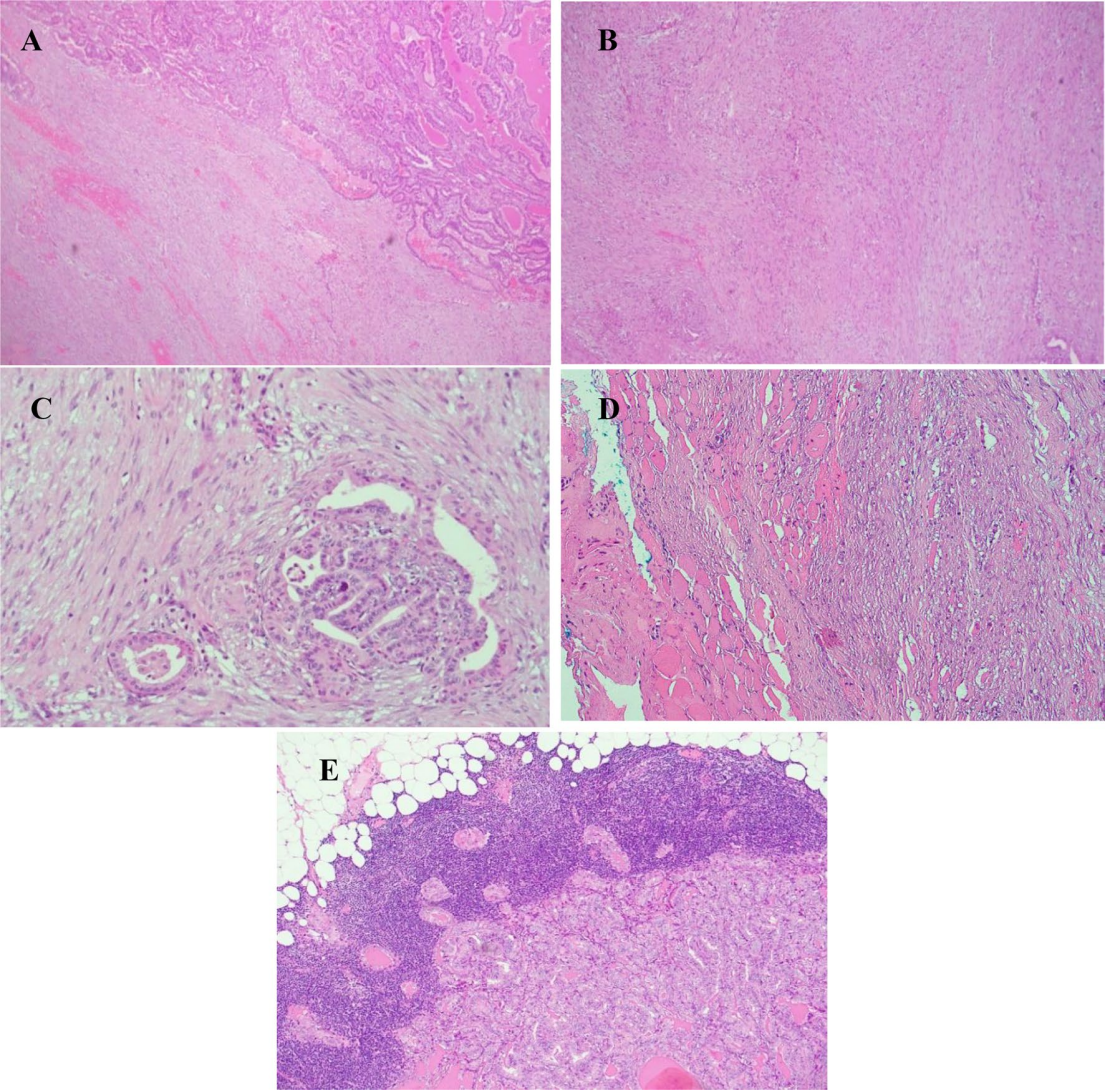
Sections showing two tumor components. (**A**) Interface between classic PTC and desmoid-type fibromatosis mesenchymal component (hematoxylin & eosin; x40); (**B**) desmoid-type fibromatosis component with broad sweeping fascicles of mostly uniform spindle cells with admixed thin-walled elongated vessels (hematoxylin & eosin; x200); (**C**) Area of classic PTC with papillary structures of cells showing enlarged overlapping nuclei with optical clearing, grooves and nuclear membrane irregularities admixed with the stromal component (hematoxylin & eosin; x400); (**D**) desmoid-type fibromatosis component invading into strap muscle bundles (hematoxylin & eosin; x200); (**E**) Metastatic papillary thyroid carcinoma to lymph node. No desmoid-type fibromatosis component is present in the metastatic deposit (hematoxylin-eosin, x40)

Histologic sections showed the tumor consisted of two components – a mesenchymal spindle cell component (comprising 60–70% of the tumor mass, approximately 3.9 to 4 cm) and an epithelial component (comprising 30% of the tumor mass, approximately 1.7 cm) The spindle cell mesenchymal component consisted of irregular broad fascicles of spindle cells lying in a vascularized fibrotic to slightly fibro-myxoid background morphologically resembling desmoid-type fibromatosis. The spindle cells had elongated, bland nuclei with fine chromatin and small, distinct nucleoli. Mitotic figures were absent. The admixed epithelial areas were comprised of classic subtype of papillary thyroid carcinoma - papillary and rare glandular structures lined by cuboidal to columnar cells with optically clear nuclei, nuclear overlapping and enlargement, nuclear grooves and occasional intranuclear inclusions (Fig. 3).

**Fig. 4:**
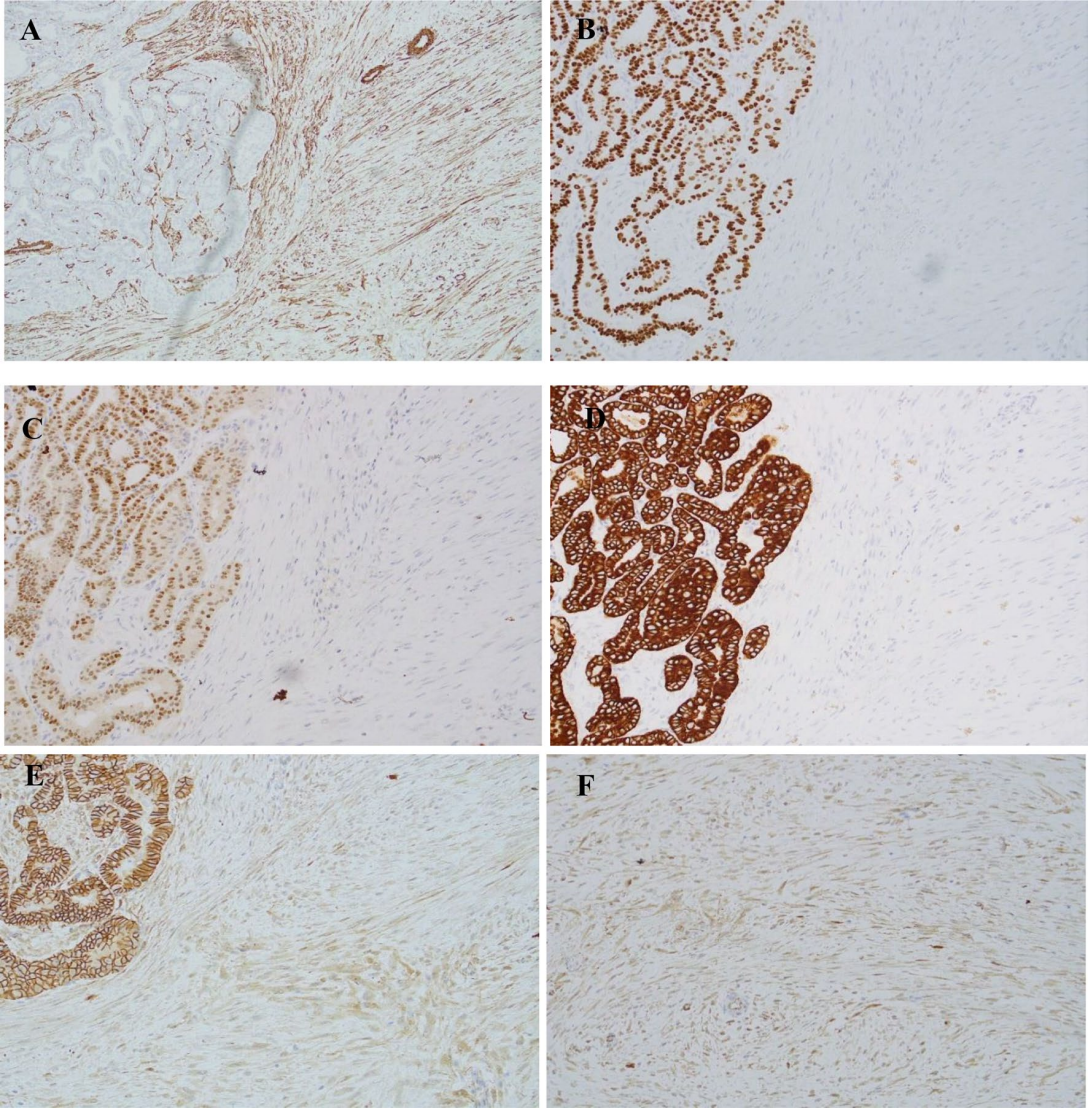
Immunohistochemical studies. (**A**) Stromal component shows diffuse staining for smooth muscle actin (SMA) while the PTC component is negative. (**B**, **C**, and **D**) PTC component with strong positive staining for TTF-1, PAX-8 and CKAE1/AE3 respectively while the stromal component is negative for all three markers (x200). (**E**) Immunohistochemical staining for β-catenin. Normal membranous staining present in the PTC component while foci of aberrant nuclear and cytoplasmic reactivity are seen in the stromal component (x200). (**F**) Aberrant nuclear β-catenin staining in desmoid-type fibromatosis stromal component (x400)

Immunohistochemical analysis was performed on formalin-fixed, paraffin-embedded tissue sections. The classic papillary carcinoma component was positive for CKAE1/AE3, PAX-8, and TTF-1 and negative for smooth muscle actin (SMA) (Fig. 4). The stromal component was positive for SMA, focally positive for desmin, and negative for CKAE1/AE3, PAX-8 and TTF-1. A key immunomarker was β-catenin, which showed patchy aberrant nuclear staining in the mesenchymal spindle cell component while the papillary carcinoma component showed normal membranous expression (Fig. 4). These immunostaining results in conjunction with the morphology helped secure the diagnosis of PTC admixed with a desmoid-type fibromatosis component. A final diagnosis of papillary thyroid carcinoma with fibromatosis/fasciitis-like/desmoid-type stroma was rendered.

The mesenchymal/fibromatosis-like component invaded into strap muscle fibers and extended to the anterior and posterior margins of the right lobe. A total of five lymph nodes were identified and two were positive for metastatic papillary carcinoma (the largest deposit was 0.3 cm with no extra nodal extension). The lymph nodes did not contain any of the mesenchymal/fibromatosis-like component (Fig. 3). According to the America Joint Commission on Cancer (AJCC 8th edition), the case was pathologically staged as pT3b, pN1a given the presence of strap muscle invasion and one involved level 6 lymph node.

Next generation sequencing (NGS) was performed on formalin-fixed paraffin-embedded tissue blocks to assess for specific mutations within the PTC component and the mesenchymal/fibromatosis-like component. NGS revealed *BRAFV600E* point mutation within the PTC epithelial component and a *CTNNB1* (β-catenin) point mutation (c.133T > C nucleotide change resulting in p.S45P amino acid change) in the mesenchymal component. These results helped further ascertain and confirm the histopathologic diagnosis.

Clinically, the patient was classified into a high-risk disease category. The treatment included adjuvant high-dose radioactive iodine ablative therapy. After 6 months offollow-up, whole body single-photon emission computed tomography (SPECT) revealed no clinical evidence of local, nodal or metastatic disease.

## Discussion

PTC with fibromatosis/fasciitis-like/desmoid-type stroma (PTC-F/FL/DTS) has been described as early as 1991 with the terminology of PTC with NFS (nodular fasciitis-like stroma) and later described as PTC with FMS (fibromatosis-like stroma) [1, 2]. This tumor subtype is extremely rare and poses diagnostic and management challenges. This is in part due to the extremely limited number of cases reported in the literature ( approximated incidence range is between 0.03 and 0.5% of all PTC cases). There is a slightly higher prevalence of this tumor subtype in women compared to men [3]. The precise etiology of PTC-F/FL/DTS remains elusive in most cases. Certain factors have been proposed as playing a role in the development of this tumor subtype including, but not limited to, surgical trauma, neoplastic infiltration by tumor cells inducing an exaggerated stromal/mesenchymal response, and influence by estrogen receptors, thereby raising the possibility of a hormone-mediated pathway in certain cases [4].

The variety of previous terms used to describe these lesions including PTC with nodular fasciitis like-stroma (PTC-NFS) and PTC with desmoid-type fibromatosis (PTC-DTF), reflects the somewhat heterogeneous nature of the associated mesenchymal components. It is difficult to determine with certainty if there are differences in biologic behavior between lesions with a nodular fasciitis-like component versus those with a desmoid-type fibromatosis component. Nodular fasciitis is a self-limited process associated with *USP6* gene rearrangements while desmoid fibromatosis is a more aggressive and infiltrative process associated with *CTNNB1* mutations [5]. Given the differences in biologic behavior of the extrathyroidal counterparts of these two spindle cell processes, an argument could be made for exact subtyping of the stromal component. However, the most recent WHO classification for Thyroid Neoplasms adopts a more unified approach and designates all of these cases as PTC with fibromatosis/fasciitis-like/desmoid-type stroma (PTC-F/FL/DTS).

With regards to clinical investigations, imaging techniques such as computed tomography (CT) may reveal a large, irregular mass with invasive growth into surrounding soft tissues and muscle bundles. However, distinguishing processes such as fibromatosis from infiltrative thyroid cancer based on imaging studies alone can be difficult.

Much like our case, prior literature highlights that fine needle aspiration cytology diagnosis of PTC-F/FL/DTS can be challenging. There is recent literature that describes both components (PTC epithelial component and aggregates of spindle cells) being present on cytologic slides after fine needle aspiration [6]. The diagnosis in that paper was fully achieved on subsequent resection tissue with appropriate ancillary studies. Our case differed from this in that ours had aggregates of atypical appearing epithelial cells and spindle cells but with no overt cytologic features of PTC; hence the more descriptive interpretation of malignant neoplasm, difficult to characterize with a suggestion of possible medullary carcinoma. Previous reports discuss a variety of interpretations on FNAC specimens including suspicious for malignancy, benign processes such as schwannoma or fibroma, and in some instances that sampled mesenchymal cells were considered irrelevant to the diagnosis [7]. In one prior case, the sampled mesenchymal component obscured the neoplastic nature of the tumor [8]. Similar to our case, it is possible that the presence of a prominent mesenchymal component may adversely impact sampling adequacy leading to a variety of interpretations.

Although the size of the PTC component in PTC-F/FL/DTS is usually small, including our case, such cases often present with extrathyroidal extension and lymph node metastases. In some instances, lymph nodes containing metastases from PTC also contain the stromal/mesenchymal component. Distant metastasis to other organ sites has not been reported for PTC-F/FL/DTS. Recent studies suggest that positive margins do not always correlate with recurrence. There is a paucity of information regarding ideal treatment of this tumor subtype due to its relative rarity. It appears that the radioactive iodine (RAI) is ineffective in the desmoplastic component, and current options include treating the patient per the pT stage as conventional PTC and optionally treating the desmoplastic component similar to extrathyroidal desmoid tumors [9]. Surgical excision, including total thyroidectomy with clear margins, represents the primary treatment modality to minimize tumor recurrence. Iodine-131 (RAI therapy), a metabolic radiotherapeutic intervention, may be used to target the carcinoma component, although its efficacy against the mesenchymal component remains limited as it lacks thyroglobulin expression. The recurrence rate widely varies with some reports documenting rare recurrences while others report a range of 20–45% [10, 11]. Additional treatment modalities such as anti-hormonal therapy, non-steroidal anti-inflammatory drugs, tyrosine kinase inhibitors, and chemotherapy may be indicated particularly as extra-thyroidal desmoid tumors can often respond to some of these therapies. Earlier studies have demonstrated favorable outcomes with low-dose methotrexate and vinblastine chemotherapy, resulting in a 10-year relapse-free survival rate of 70% [3–10, 12]. The biologic behavior of desmoid fibromatosis can be unpredictable and rare cases can sometimes show regression. Historically, achieving clear surgical margins was considered to be optimal care. However, the literature details that recurrences do not always correlate with margin status. Additionally, multiple surgeries to achieve clear margins comes with additional risk and morbidity. This has recently led to the development of a careful observation approach, particularly in those patients without symptoms [13–15]. Studies have highlighted that the presence of distinct *CTNNB1* mutations, such as the Ser45Phe mutation, in desmoid-type fibromatosis may be associated with increased disease recurrence in other body sites [16, 17].

Our case, similar to prior reports, demonstrated aberrant nuclear β-catenin expression by immunohistochemistry and also showed a *CTNNB1* point mutation, both of which are frequently observed in desmoid-type fibromatosis. This mutation may contribute to adverse behavior and aggressiveness in select cases. Previous studies have shown that the *CTNNB1* gene (encodes β-catenin) plays a key role in the Wnt-signal pathway. Mutations of this gene lead to retention of β-catenin via impaired degradation. As a result, β-catenin enters the cell nucleus, leading to increased transcription of downstream target genes and subsequent increased cell proliferation. This ultimately has an oncogenic effect and can lead to the development of a variety of associated tumors such as desmoid-type fibromatosis [11]. The occurrence of distinct *CTNNB1* mutations, such as the Ser45Phe mutation, may be associated with increased disease recurrence. However, larger studies with clinical follow-up is critical to evaluate the prognostic impact of these aforementioned specific mutations. These findings hold potentially significant clinical implications, given the differing behaviors of nodular fasciitis (often spontaneous regression) versus the more aggressive course seen in fibromatosis [12, 18, 19]. The impact or significance of the presence of *CTNNB1* mutation in this extremely rare tumor subtype has yet to be determined.

The differential diagnosis for PTC-F/FL/DTS includes other thyroid malignancies, spindle cell processes and non-neoplastic conditions. Classic subtype of papillary thyroid carcinoma should be considered as this subtype can often have a variably prominent desmoplastic or fibrotic background. In such cases with a prominent fibrotic or desmoplastic background, careful examination is suggested to help distinguish it from the broad, elongated sweeping fascicles of fibroblastic and myofibroblastic cells seen in desmoid-type fibromatosis. Inflammatory conditions such as IgG4-mediated thyroiditis and fibrosing lymphocytic thyroiditis can have some overlapping morphologic features with PTC-F/FL/DTS. Careful gross and microscopic evaluation of the specimen including detailed study of the relevant clinical history, laboratory findings, and imaging features can be helpful. Other differential considerations include other benign processes such as extra-thyroidal soft tissue nodular fasciitis or desmoid-type fibromatosis involving or surrounding the thyroid, and even the very rare primary intrathyroidal desmoid-type fibromatosis [20]. These lesions can be morphologically and immunohistochemically difficult to distinguish from the mesenchymal component of PTC-F/FL/DTS as they are both myofibroblastic/fibroblastic processes with similar morphologies and immunoprofiles. The bulk or epicenter of the spindle cell/mesenchymal component within the thyroid helps exclude extrathyroidal fibromatosis and nodular fasciitis. However, in the context of intrathyroidal processes, the presence of admixed PTC is critical to securing a diagnosis of PTC-F/FL/DTS.

It is also important to differentiate this rare tumor subtype from more aggressive malignancies such as anaplastic thyroid carcinoma, specifically the sarcomatoid/spindle cell pattern. Anaplastic thyroid carcinoma is a highly aggressive and rapidly growing thyroid malignancy that often presents with a large, infiltrative mass and often invades surrounding structures. It exhibits marked cellular pleomorphism, multinucleation, and necrosis and can often show undifferentiated morphology on histological examination. In a subset of anaplastic carcinoma cases, immunohistochemical markers such as p53, PAX-8, keratins can be positive which can be helpful in distinguishing anaplastic carcinoma from the spindle cell/fibromatosis component of PTC-F/FL/DTS [21]. Additionally, the presence of B-catenin nuclear staining can help confirm and distinguish fibromatosis from a spindle cell/sarcomatoid pattern of anaplastic carcinoma. Clinical factors are also critical in this differential distinction as in our case, the patient is young and lacks clinical features typically associated with anaplastic carcinoma such as a rapidly growing mass with compression symptoms. Molecular testing for mutations commonly associated with anaplastic carcinoma, such as *TP53* mutations, can aid in the differential diagnosis.

Therefore, a comprehensive evaluation of the patient’s clinical history, imaging findings, and pathological features is crucial for accurate diagnosis and appropriate management.

## Conclusions

We present a case of papillary thyroid carcinoma with fibromatosis/fasciitis-like/desmoid-type stroma with a discussion of important differential considerations and also highlight the importance of keeping this rare subtype in mind when evaluating papillary carcinomas with a prominent spindled or fibrotic appearing background or other spindle cell processes of the thyroid. Our report adds additional data and information to the slowly growing list of reported cases of this subtype in the literature. Awareness of this tumor subtype is critical for pathologists for prevention of misclassification, particularly given its peculiar morphologic features and extremely rare frequency. Our case also highlights the challenges associated with attempting to establish a diagnosis based on imaging and FNA-Cytology. This could in part be due to the difficulty in obtaining an adequate aspirate sample if the stromal component comprises the bulk of the mass (as was in our case). Additionally, the cytologic features of any aspirated epithelial component may be obscured given their admixed nature with the stromal component. Our report highlights the critical importance of careful and rigorous histopathologic evaluation and judicious use of ancillary immunohistochemical and molecular studies to establishing the diagnosis and thus providing clinicians with the optimal data which will aid in delivering the most effective management and treatment plan possible to patients.

## Data Availability

No datasets were generated or analysed during the current study.

## References

[1] Chan JK, Carcangiu ML (1991) Rosai Papillary carcinoma of thyroid with exuberant nodular fasciitis-like stroma. Rep Three Cases Am J Clin Pathol 95:309–314 10.1093/ajcp/95.3.309 1996541

[2] Mizukami Y, Nonomura A, Matsubara F, Michigishi T, Ohmura K (1992) Hashimoto Papillary carcinoma of the thyroid gland with fibromatosis-like stroma histopathology. 20:355–357 10.1111/j.1365-2559.1992.tb00994.x 1577414

[3] Toniato A, Brusoni M, Mirabella M et al (2023) Papillary thyroid carcinoma with fibromatosis-like stroma: a case report and review of the literature. BMC Endocr Disord 23:80 37060011 10.1186/s12902-023-01337-y PMC10103504

[4] Wu Z, Chu X, Fan S, Meng X, Xu C (2013) Papillary thyroid carcinoma with fibromatosislike stroma: a case report and review of the literature. Oncol Lett 5(1):215–217 23255922 10.3892/ol.2012.993 PMC3525473

[5] Patel N, Chrisinger J, Demicco E et al (2017) *USP6* activation in nodular fasciitis by promoter-swapping gene fusions. Mod Pathol 30:1577–1588 28752842 10.1038/modpathol.2017.78

[6] Juhlin CC, Hysek M, Stenman A, Zedenius J (2022) Papillary thyroid carcinoma with desmoid-like Fibromatosis: double trouble? Endocr Pathol 33(4):525–527. 10.1007/s12022-022-09735-z Epub 2022 Oct 15. PMID: 36242758; PMCID: PMC9712337) 36242758 10.1007/s12022-022-09735-z PMC9712337

[7] Takada N, Hirokawa M, Ito M, Ito A, Suzuki A, Higuchi M, Kuma S, Hayashi T, Kishikawa M, Horikawa S et al (2017) Papillary thyroid carcinoma with desmoid-type fibromatosis: a clinical, pathological, and immunohistochemical study of 14 cases. Endocr J 64:1017–1023 28794344 10.1507/endocrj.EJ17-0242

[8] Roukain A, La Rosa S, Bongiovanni M, Nicod Lalonde M, Cristina V, Montemurro M, Cochet S, Luquain A, Kopp PA, Sykiotis GP (2021) Papillary thyroid carcinoma with desmoid-type fibromatosis: review of published cases. Cancers (Basel) 13(17):4482. 10.3390/cancers13174482 PMID: 34503292; PMCID: PMC8430917 34503292 10.3390/cancers13174482 PMC8430917

[9] Yang YJ, LiVolsi VA, Khurana KK (1999) Papillary thyroid carcinoma with nodular fasciitis-like stroma. Pitfalls in fine-needle aspiration cytology. Arch Pathol Lab Med 123(9):838–841 10458836 10.5858/1999-123-0838-PTCWNF

[10] Zand V, Moghimi M, Sadeghi E, Kamal P, Vaziribozorg S (2022 Spring) Papillary thyroid carcinoma with nodular Fasciitis-Like Stroma in a 28-Year-old patient. Iran J Pathol 17(2):225–228 Epub 2022 Mar 8 10.30699/IJP.2022.139405.2525 PMC9013871 35463724

[11] Roth EM, Courtney E, Barrows M, Nishino B, Sacks P-O, Hasselgren (2019) James. Papillary thyroid cancer with extrathyroidal extension of desmoid-type fibromatosis. A case report of an aggressive presentation of an uncommon pathologic entity. Int J Surg Case Rep 63:5–9 31499326 10.1016/j.ijscr.2019.08.001 PMC6734537

[12] Rebecchini C, Nobile A, Piana S et al (2017) Papillary thyroid carcinoma with nodular fasciitis-like stroma and β-catenin mutations should be renamed papillary thyroid carcinoma with desmoid-type fibromatosis. Mod Pathol 30:236–245 27713418 10.1038/modpathol.2016.173

[13] Salas S, Dufresne A, Bui B, Blay JY, Terrier P, Ranchere-Vince D, Bonvalot S, Stoeckle E, Guillou L, Le Cesne A, Oberlin O, Brouste V, Coindre JM (2011) Prognostic factors influencing progression-free survival determined from a series of sporadic desmoid tumors: a wait-and-see policy according to tumor presentation. J Clin Oncol 29(26):3553–3558 21844500 10.1200/JCO.2010.33.5489

[14] Colombo C, Foo WC, Whiting D, Young ED, Lusby K, Pollock RE, Lazar AJ, Lev D (2012) FAP-related desmoid tumors: a series of 44 patients evaluated in a cancer referral center. Histol Histopathol 27(5):641–649 22419028 10.14670/HH-27.641

[15] Kasper B, Baumgarten C, Garcia J, Bonvalot S, Haas R, Haller F, Hohenberger P, Penel N, Messiou C, van der Graaf WT, Gronchi A, Desmoid Working Group (2017) An update on the management of sporadic desmoid-type fibromatosis: a European Consensus Initiative between Sarcoma patients EuroNet (SPAEN) and European Organization for Research and Treatment of Cancer (EORTC)/Soft tissue and Bone Sarcoma Group (STBSG). Ann Oncol 28(10):2399–2408 28961825 10.1093/annonc/mdx323 PMC5834048

[16] Colombo C, Miceli R, Lazar AJ, Perrone F, Pollock RE, Le Cesne A, Hartgrink HH, Cleton-Jansen AM, Domont J, Bovée JV, Bonvalot S, Lev D, Gronchi A (2013) CTNNB1 45F mutation is a molecular prognosticator of increased postoperative primary desmoid tumor recurrence: an independent, multicenter validation study. Cancer 119(20):3696–3702 23913621 10.1002/cncr.28271

[17] Lazar AJ, Tuvin D, Hajibashi S, Habeeb S, Bolshakov S, Mayordomo-Aranda E, Warneke CL, Lopez-Terrada D, Pollock RE, Lev D (2008) Specific mutations in the beta-catenin gene (CTNNB1) correlate with local recurrence in sporadic desmoid tumors. Am J Pathol 173(5):1518–1527 18832571 10.2353/ajpath.2008.080475 PMC2570141

[18] Huang H, Li L, Liu X, Zhao L, Cui Z, Zhang R, Chen S (2023) Papillary thyroid carcinoma with desmoid-type fibromatosis: the clinicopathological features with characteristic imaging and molecular correlation requiring comprehensive treatment. Hum Pathol 136:84–95 37019411 10.1016/j.humpath.2023.03.019

[19] Suster D, Michal M, Nishino M, Piana S, Bongiovanni M, Blatnik O, Hájková V (2020) Nikola Ptáková, Michal, and Saul Suster. Papillary thyroid carcinoma with prominent myofibroblastic stromal component: clinicopathologic, immunohistochemical and next-generation sequencing study of seven cases. Mod Pathol 33(9):1702–1711 32291398 10.1038/s41379-020-0539-7

[20] Singh BK, Chumber S, Rathore YS, Agarwal S, Rastogi S, Surabhi V (2023) Recurrent desmoid fibromatosis of the thyroid gland: a diagnostic challenge. J ASEAN Fed Endocr Soc 38(1):120–124 37252415 10.15605/jafes.038.01.16 PMC10213382

[21] Xu B, Ghossein RA (2023) Advances in thyroid Pathology: high Grade Follicular Cell-derived thyroid carcinoma and anaplastic thyroid carcinoma. Adv Anat Pathol 30(1):3–10 36306188 10.1097/PAP.0000000000000380

